# Decoding Light-Spreading Intensity Effects on the Sensory Quality and Volatile Compounds of Green Tea: An Integrated GC-E-Nose and Targeted Metabolomics Analysis

**DOI:** 10.3390/foods14081313

**Published:** 2025-04-10

**Authors:** Qiwei Wang, Jiajing Hu, Jiahao Tang, Xianxiu Zhou, Haibo Yuan, Yongwen Jiang, Jialing Xie, Yanqin Yang

**Affiliations:** 1 National Key Laboratory for Tea Plant Germplasm Innovation and Resource Utilization, Tea Research Institute, Chinese Academy of Agricultural Sciences, Hangzhou 310008, China; wangqiwei@tricaas.com (Q.W.); 15926789897@163.com (J.H.); tangjiahao@tricaas.com (J.T.); zhouxianxiu@tricaas.com (X.Z.); 192168092@tricaas.com (H.Y.); jiangyw@tricaas.com (Y.J.); 2 State Key Laboratory of Tea Plant Biology and Utilization, Anhui Agricultural University, Hefei 230036, China; 3 College of Tea Science, Yunnan Agricultural University, Kunming 650201, China; 4 Hezhou Agriculture and Rural Affairs Bureau, Hezhou 542800, China

**Keywords:** light intensity, green tea, GC-E-Nose, GC-MS/MS, volatile compounds

## Abstract

Spreading, the preliminary step in the production of green tea, is crucial for achieving superior tea quality. This study investigated the effects of spreading on the sensory quality and volatile compounds in green tea under varying intensities of yellow light, employing GC-E-Nose and targeted metabolomics. A notable improvement in overall sensory quality was noted in tea samples subjected to a higher intensity of 6000 Lux, which was characterized by a delightful floral fragrance. In total, 70 volatile compounds were successfully identified, with 61 volatiles detected across all five light intensities. Moreover, 21 pivotal odorants featuring odor activity value (OAV) levels higher than one were determined, among which *β*-ionone, *β*-damascenone, linalool, (*E*, *Z*)-2,6-nonadienal, and phenylethyl alcohol exhibited particularly high OAVs. Correlation analysis indicated that phenylethyl alcohol, linalool, and citral exhibited robust positive correlations with the majority of key odorants, suggesting their vital contribution towards aroma enhancement. These findings offer novel insights into the regulation of tea aroma through the manipulation of light intensity during the processing of green tea.

## 1. Introduction

Tea is recognized globally as the second most prevalent beverage after water. Depending on the manufacturing techniques and fermentation degrees, tea is primarily classified into six major categories. Among them, green tea stands as the most abundant tea type in terms of production and consumption in China, gaining widespread acceptance among consumers due to its extensive health benefits and distinctive aromas. Representative fragrances, such as floral aroma and chestnut-like aroma, are highly favored by consumers due to their delightful flavor characteristics [1].

Aroma is a crucial criterion in the assessment of tea quality. Essentially, it involves the intricate interplay of diverse concentrations and types of volatile substances. Currently, there are approximately 700 volatile components identified in tea, with more than 100 volatile components primarily found in fresh leaves [2]. The remaining volatile compounds are predominantly produced through diverse processing methods, suggesting that variations in these techniques can significantly affect the aroma of tea [3]. Spreading is an essential process for producing high-quality green tea. During the leaf-spreading process, progressive water loss induces cytoplasmic volume contraction. Endogenous enzyme activity may increase due to the concentration effect—a decrease in water content leads to higher effective concentrations of both enzymes and substrates, consequently accelerating the rate of enzymatic reactions [4]. This subsequently promotes the degradation of large molecules like chlorophyll, proteins, fatty acids, and polysaccharides in fresh leaves and generates secondary metabolites, which significantly affect the aroma, taste, and color of green tea [5]. The formation of a favorable aroma type in premium green tea is intimately associated with the spreading process [6,7].

Light, as a critical environmental modulator, exerts profound regulatory effects on the metabolic pathways of *Camellia sinensis*. In addition, recent advances have revealed the practical applications of light-mediated interventions in postharvest tea processing. For instance, controlled light exposure during black tea withering has been demonstrated to enhance the aroma profiles by modulating enzymatic activities and gene expression patterns [8,9]. Notably, emerging evidence demonstrates that yellow light irradiation during spreading significantly improves tea quality compared to red light and orange light treatments [10,11]. The superior effect of yellow light may result from its regulation of secondary metabolite biosynthesis related to tea flavor and aroma [8,12]. Nevertheless, to our knowledge, the effects of spreading on the sensory attributes and volatile compounds in green tea under yellow light with different intensities (YLDI) have yet to be thoroughly and comprehensively explored, which hinders the optimization of light-mediated regulating strategy in green tea production.

The traditional assessment method for tea quality predominantly depends upon expert sensory analysis, which is susceptible to environmental factors and exhibits individual variability. As an important complement to conventional evaluation, the electronic nose is conceptualized on the principle of bionics and emulates the human olfactory system through an array of sensors, possessing the advantages of no pretreatments and rapid analysis. However, this technique is susceptible to sensor drift and contamination. In contrast to traditional electronic noses, the gas chromatography-based electronic nose (GC-E-Nose) is an odor analysis technology based on gas chromatography principles, facilitating the separation of odor substances through two distinct polarity chromatographic columns [13]. Owing to its distinguishing features of excellent sensitivity and swift analysis speed, GC-E-Nose is routinely utilized in profiling the volatile fingerprints of tea [14].

Metabolomics, as a powerful analytical technique, facilitates the comprehensive exploration of small molecule metabolites through high-throughput detection and data analysis. This approach provides robust data support for the enhancement and refinement of food flavor. Depending on the research objectives and applications, metabolomics is categorized into targeted metabolomics and untargeted metabolomics. The latter is the prevalent strategy for metabolic profiling, providing comprehensive data for unknown metabolites. Nevertheless, this approach is not suitable for the quantitative evaluation of metabolites, necessitating the incorporation of high-resolution mass spectrometry to facilitate the identification and characterization of unidentified metabolites. Targeted metabolomics, also known as quantitative metabolomics, primarily focuses on specific metabolites or metabolic pathways. Gas chromatography-mass spectrometry (GC-MS) serves as the predominant method for targeted analysis of volatile compounds, particularly under the multiple reaction monitoring (MRM) mode. In other words, the triple quadrupole tandem mass spectrometer (MS/MS) provides enhanced sensitivity and superior specificity by detecting precursor ions and characteristic product ions of metabolites. Given these excellent performances, gas chromatography-tandem mass spectrometer (GC-MS/MS) has been extensively utilized in targeted analysis of heterocyclic compounds such as pyrazines, pyrroles, and furans present in green tea [15,16].

The current investigation thoroughly examined the impact of spreading under YLDI on the sensory quality and volatile compounds of green tea, utilizing a combination of GC-E-Nose and targeted GC-MS/MS. Our results are expected to offer a theoretical foundation for the enhancement of processing technologies, thereby facilitating the production of premium-grade green tea more efficiently.

## 2. Materials and Methods

### 2.1. Materials and Reagents

The main materials utilized for this study included headspace vials (20 mL; Agilent Technologies Inc., Palo Alto, CA, USA), a manual solid-phase microextraction (SPME) handle, and a DVB/CAR/PDMS fiber (Bellefonte, PA, USA). The reagent used in this study was termed as purified water (Hangzhou Wahaha Group Co., Ltd., Hangzhou, China). The standard reference compounds employed in this study are detailed in Table S1.

### 2.2. Preparation of Tea Samples

Young shoots of *Camellia sinensis* cultivar ‘Longjing Changye’, comprising a single bud and a single leaf, were collected from Shengzhou City in Zhejiang Province. The intricate processing involved several stages, including spreading, fixation, rolling, first drying, and second drying, with the specific processing parameters illustrated in Figure S1. During the spreading stage, tea leaves were exposed to yellow light of varying intensities (500 Lux, 1000 Lux, 2000 Lux, 4000 Lux, and 6000 Lux). The equipment for light spreading used in this study was an artificial climatic chamber (PRX-4500, Ningbo Prant Instrument Co., Ltd., Ningbo, China). To ensure reproducibility, three independent processing batches (including experimental and validation groups) were performed following identical protocols in April, June, and October 2023. The completed tea samples were preserved at a temperature as low as −20 °C for subsequent analysis.

### 2.3. Sensory Evaluation

The sensory evaluation was in accordance with the Chinese standard, namely, GB/T 23776-2018 [17]. Ethical permission was not deemed necessary for this study. A panel of six experienced evaluators, who provided their consent to participate in the sensory assessment and to utilize their information, assessed the tea samples subjected to YLDI. The evaluation protocol was as follows: Initially, the tea samples were positioned on a tea tray, and the appearance attribute, including color, tightness, and uniformity, was assessed. Subsequently, 3 g of the tea sample were steeped in 150 mL of boiling water for 4 min. The color and taste attributes were assessed by filtering the tea infusion into an assessment bowl. The aroma attribute was evaluated using hot, warm, and cold sniffing to confirm the type (such as chestnut-like aroma, floral aroma, fresh scent, or their complex fragrance types), intensity, and persistence of the aroma. Lastly, the infused leaf was evaluated. The comprehensive quality was determined utilizing a 100-point scale, with the following weightings assigned to each attribute: appearance (25%), infusion color (10%), aroma (25%), taste (30%), and infused leaf (10%).

### 2.4. GC-E-Nose Analysis

The GC-E-Nose, acquired from Alpha M.O.S. (Toulouse, France), was employed to investigate the volatile profiles of tea samples exposed to YLDI. The operational parameters were subtly adjusted by referring to earlier studies [18]. The specific parameters were as follows: 0.5 g of tea samples were placed into a hermetically sealed headspace vial (20 mL). Then, the sample vial was positioned in the designated tray location for extraction. The incubation temperature and duration of the GC-E-Nose were set to 60 °C and 20 min, respectively. The injection port temperature was adjusted to 200 °C, with an injection volume of 5000 μL. An integrated odor concentrator, Tenax TA trap, was employed to enrich the volatiles at 20 °C, with an adsorption time of 27 s. The temperature profiles of the two distinct columns (MXT-5 and MXT-1701 columns) were initially maintained at 50 °C (for a duration of 5 s), followed by a gradual increase at a rate of 0.1 °C/s until reaching 80 °C, then further increased at a rate of 0.4 °C/s until 250 °C was achieved, after which it was held for 10 s. For the flame ionization detector, both were operated at 260 °C.

### 2.5. HS-SPME Coupled to GC-MS/MS Analysis

The HS-SPME procedures involved placing tea samples (0.5 g) and purified water (5 mL) within a 20-mL headspace vial. The DVB/CAR/PDMS fiber was used for the enrichment of volatile substances. The recommended conditions for the incubation were at an optimal temperature of 60 °C for 1 h. Following this, the fiber underwent desorption for a period of 5 min, with the injector temperature maintained at 230 °C.

The profiling of volatile compounds involved the application of an Agilent 7890A gas chromatograph integrated with an Agilent 7000C series triple quadrupole system (Agilent Technologies Inc., Palo Alto, CA, USA). The operational parameters for GC-MS/MS were established following the methodology outlined by Hu et al. [19]. A DB-5MS capillary column (30 m × 0.25 mm × 0.25 μm) was utilized for the separation of the volatile compounds. The oven temperature was initially set at 40 °C for a duration of 5 min, followed by a gradual increase at a rate of 4 °C/min until reaching 160 °C, where it was maintained for an additional 5 min. The carrier gas of helium was at a rate of 1.0 mL/min. The collision gas, high-purity nitrogen (99.999%), was supplied at a rate of 1.5 mL/min, with the quench gas of helium at a rate of 2.25 mL/min. A splitless injection mode was employed for this analysis. The operational temperature for the ion source remained steady at 230 °C, concurrently ensuring that the transfer line temperature was held at 270 °C. The mass spectrometer operated in MRM mode, ensuring that the mass resolutions for MS1 and MS2 were optimized to their respective widest and unity settings.

The identification of volatile compounds was conducted through a comparative analysis of the retention time and mass spectra against the corresponding reference standards. The accurate quantification of these volatiles was performed using the external standard method [11]. The calibration curves were developed by preparing a series of calibration solutions with different established gradients. Comprehensive details regarding the calibration information are provided in Table S2.

### 2.6. Odor Activity Value (OAV) Analysis

OAV is an essential tool for assessing the individual contribution of compounds. It is calculated by utilizing the ratio between the specified concentration of each constituent and its corresponding threshold level in water obtained from the literature [19]. Compounds with an OAV of one or higher are recognized as potential contributors to the overall aroma profile.

### 2.7. Statistical Analysis

All the experiments were conducted in triplicate, and the data were reported as the mean and standard deviation. Graphic representations, including Venn diagrams and bar charts, were performed utilizing Origin 20 software (OriginLab Corporation, Northampton, MA, USA). Partial least squares discrimination analysis (PLS-DA), metabolic trajectory mapping, and orthogonal PLS-DA (OPLS-DA) were conducted using SIMCA-P 14 software (Umetrics, Umea, Sweden). Statistical significance between different samples was determined using one-way analysis of variance (ANOVA), followed by Tukey’s multiple comparison test (*p* < 0.05), conducted with SPSS 20 software (SPSS Inc., Chicago, IL, USA). The hierarchical clustering (HCL) heat maps depicting key aroma compounds and their correlation analysis were generated using the Chiplot online tool (https://www.chiplot.online/#Heatmap accessed on 6 May 2024).

## 3. Results and Discussion

### 3.1. Sensory Evaluation Analysis on Tea Samples Exposed to YLDI

The sensory quality of tea samples subjected to YLDI are detailed in Table S3 through systematic sensory analysis. With respect to the taste attribute, the tea samples exposed to 6000 Lux, 1000 Lux, and 4000 Lux attained scores exceeding 90. Notably, the samples under 6000 Lux attained the highest score of 93, exhibiting a fresh taste with floral flavor. Regarding the aroma attribute, all five tea samples scored above 90, further confirming that yellow light significantly improved the aroma profile of green tea. Specifically, the tea samples under 500 Lux and 2000 Lux predominantly displayed a tender chestnut-like aroma, whereas the tea samples under 1000 Lux demonstrated a chestnut-like aroma accompanied by floral notes. The samples exposed to 4000 Lux and 6000 Lux were distinguished by a pronounced floral aroma, with the 6000 Lux samples exhibiting a particularly strong and enduring floral fragrance. According to their aroma types, the tea samples under YLDI could be categorized into three groups: tender chestnut-like aroma (500 Lux and 2000 Lux), floral aroma (4000 Lux and 6000 Lux), and chestnut-like aroma with floral aroma (1000 Lux). In terms of overall quality, the tea samples processed under 6000 Lux achieved the highest score, followed by those under 1000 Lux, 4000 Lux, 2000 Lux, and 500 Lux, respectively. These results indicate that higher light intensities are beneficial for enhancing the sensory quality of green tea, particularly in promoting the development of floral notes.

### 3.2. Analysis of the Effects of YLDI on the Volatile Fingerprints of Green Tea Using GC-E-Nose

The tea samples subjected to YLDI underwent analysis via GC-E-Nose, and the volatile fingerprints were obtained. Distinctive variations were observed in the aroma profiles of tea samples under YLDI (Figure S2). Notably, the tea samples exposed to 1000 Lux exhibited fewer volatile peaks on the MXT-1701 and MXT-5 columns compared to the other four treatments, indicating a unique aroma profile for the 1000 Lux samples. The volatile fingerprints of the 500 Lux and 2000 Lux samples were similar, with only minor differences in peak intensities, indicating comparable odor profiles. Additionally, the 6000 Lux samples exhibited lower peak intensities on the MXT-1701 column than other samples. The above results suggest that different light intensities directly influence the volatile fingerprints of green tea.

**Figure 1:**
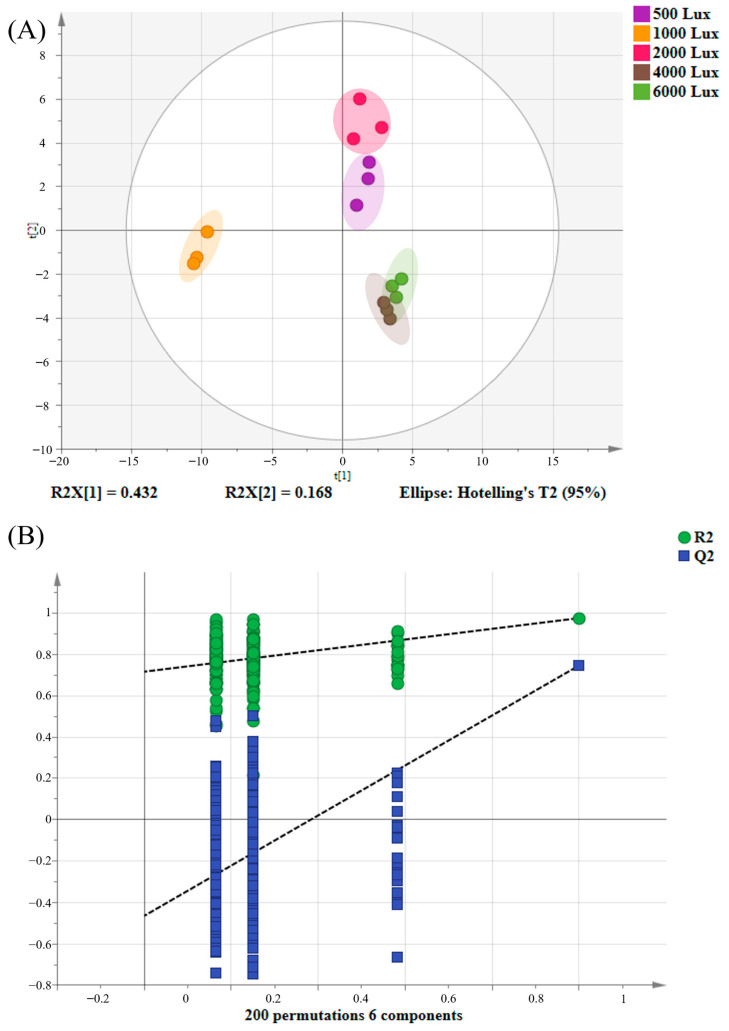
PLS-DA results of tea samples exposed to yellow light with different intensities based on GC-E-Nose. (**A**) Score plots (R^2^Y = 0.987, Q^2^ = 0.882); (**B**) Permutation tests with 200 iterations (R^2^ = 0.717, Q^2^ = −0.465).

To explore the effects of YLDI on the aroma quality of green tea, a supervised PLS-DA was employed for multivariate statistical analysis. This approach aims to construct a correlation model that correlates sample categories with metabolic expression levels [20,21]. As illustrated in Figure 1A, the tea samples subjected to 500 Lux and 2000 Lux were clustered at the first quadrant of the score plot, whereas those exposed to 4000 Lux and 6000 Lux were grouped at the fourth quadrant. The 1000 Lux samples were clustered separately on the third quadrant, aligning with the findings from sensory assessment. The model parameters were R^2^Y = 0.987 and Q^2^ = 0.882, respectively, indicating robust explanatory power and predictive ability. In addition, a permutation test was performed with 200 iterations. The parameters of R^2^ = (0.0, 0.717) and Q^2^ = (0.0, −0.465) validated the model’s reliability without overfitting (Figure 1B). In summary, GC-E-Nose serves as a complementary and validation tool for traditional sensory evaluation and can rapidly and accurately distinguish tea samples by integrating with multivariate statistical analysis.

### 3.3. Analysis of the Effects of YLDI on the Volatile Compounds of Green Tea Using GC-MS/MS

#### 3.3.1. Comparative Analysis of the Volatile Compounds in Tea Samples

**Figure 2:**
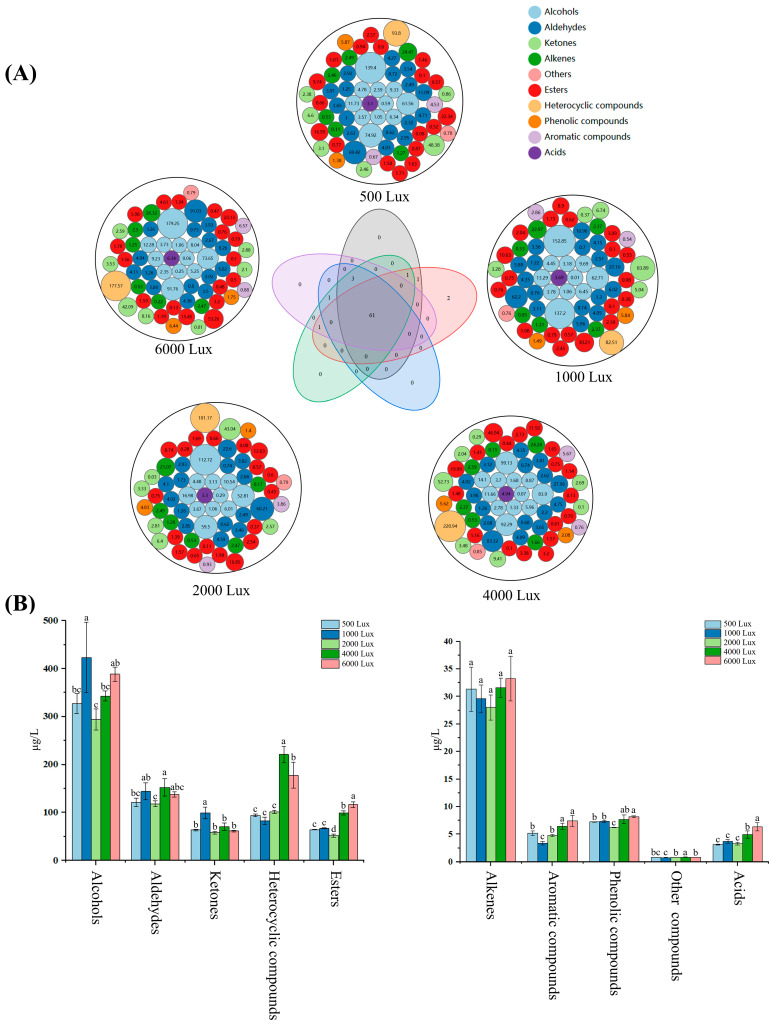
Information of volatile compounds in tea samples exposed to yellow light with different intensities based on GC-MS/MS. (**A**) Volatile compounds in tea samples under different treatments; (**B**) Content comparison of different volatile categories. Different letters indicated significant differences (*p* < 0.05).

In this study, a total of 70 volatile compounds were targeted and analyzed by GC-MS/MS, with detailed quantitative information depicted in Figure 2A and Table S4. Notably, 61 volatile compounds were consistently identified across five different light intensity conditions. In addition, certain volatile substances were detected solely under specific light intensity treatments. For instance, 3-octanone was observed solely in the samples subjected to 4000 Lux and 6000 Lux treatments; (*E*)-2-hexenal was found solely in the samples exposed to 500 Lux and 1000 Lux; *cis*-3-hexenyl salicylate and (*3Z*)-3-hexen-1-yl benzoate were detected solely in samples treated with 1000 Lux.

The effects of YLDI on different classes of volatile compounds also displayed substantial variations (Figure 2B). Alcohols reached the highest in the samples processed under 1000 Lux, significantly exceeding those under 500 Lux, 2000 Lux, and 4000 Lux (*p* < 0.05). Aldehydes attained the maximum in the tea samples processed under 4000 Lux (152.32 μg/L), followed by the samples processed under 1000 Lux and 6000 Lux, with significantly lower concentrations in the samples processed under 500 Lux and 2000 Lux (*p* < 0.05). Ketones achieved the maximum in the samples subjected to 1000 Lux (99.32 μg/L), which was significantly greater than the levels observed under other light intensities (*p* < 0.05). Heterocyclic compounds were ordered as 4000 Lux > 6000 Lux > 2000 Lux > 500 Lux > 1000 Lux, with the tea samples treated under 4000 Lux significantly higher than other treatments (*p* < 0.05). Esters and Alkenes were the most abundant in tea samples exposed to 6000 Lux. Aromatic hydrocarbons and phenolic compounds were more abundant in the 4000 Lux and 6000 Lux samples, significantly higher than other treatments (*p* < 0.05). Geranic acid exhibited the highest concentration in the 6000 Lux treated sample, significantly higher than other samples (*p* < 0.05). In summary, these results indicate that YLDI significantly influences both the numbers and concentrations of aroma components in green tea, thereby affecting the overall manifestation of the fragrance profile.

#### 3.3.2. Multivariate Statistical Analysis

**Figure 3:**
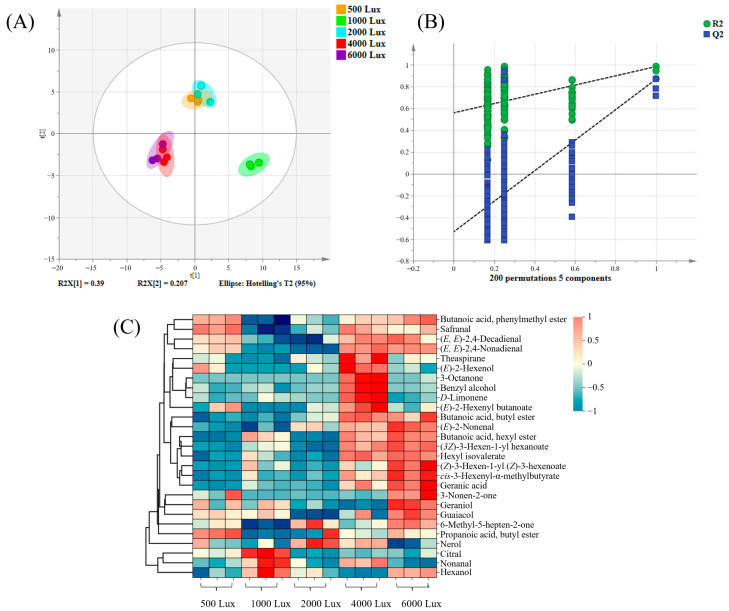
PLS-DA results of volatile compounds in tea samples exposed to yellow light with different intensities based on GC-MS/MS. (**A**) Score plots (R^2^Y = 0.982, Q^2^ = 0.896); (**B**) Permutation tests with 200 iterations (R^2^ = 0.562, Q^2^ = −0.528); (**C**) Heatmap visualization constructed with VIP > 1.

PLS-DA was employed to further analyze the volatile components obtained from GC-MS. Favorable parameters (R^2^Y = 0.982 and Q^2^ = 0.896) were attained, which suggested a robust explanatory capacity and predictive ability. As depicted in Figure 3A, a notable differentiation among the samples was observed, with the distribution patterns of tea samples subjected to five varying light intensities aligning with the findings from the GC-E-Nose analysis. Specifically, the samples exposed to 500 Lux and 2000 Lux were clustered primarily in the upper quadrant of the score plots, while those exposed to 4000 Lux and 6000 Lux were primarily located in the lower left quadrant. Conversely, samples treated with 1000 Lux were situated in the lower right quadrant. Additionally, the reliability of the PLS-DA model was assessed through 200 iterations of permutation testing, which confirmed the absence of overfitting, as depicted in Figure 3B.

To identify the key differential components in tea samples exposed to YLDI, hierarchical cluster analysis was performed. A total of 27 key volatiles were identified based on thresholds of *p* < 0.05 and variable importance in the projection (VIP) > 1. As depicted in Figure 3C, (*E*, *E*)-2,4-nonadienal, butanoic acid, phenylmethyl ester, safranal, and (*E*, *E*)-2,4-decadienal were identified as characteristic volatiles distinguishing the tea samples treated with 500 Lux from those subjected to other light intensities. Citral, nonanal, and hexanol were more abundant in the 1000 Lux samples than other treatments. Nerol exhibited a more pronounced differentiation in the 2000 Lux samples compared to other treatment conditions. Higher levels of theaspirane, (*E*)-2-hexenol, 3-octanone, benzyl alcohol, *D*-limonene, and (*E*)-2-hexenyl butanoate were attained in tea samples exposed to 4000 Lux. The contents of (*E*)-2-nonenal, butanoic acid, hexyl ester, (*3Z*)-3-hexen-1-yl hexanoate, hexyl isovalerate, (*Z*)-3-hexen-1-yl (*Z*)-3-hexenoate, *cis*-3-hexenyl-α-methylbutyrate, and geranic acid displayed elevated levels in the tea samples exposed to 6000 Lux compared to other treatments.

#### 3.3.3. OAV Analysis

It is universally acknowledged that not all volatile components exert influence on the overall profile of green tea. To screen the pivotal aroma contributors in tea samples exposed to five varying light intensities, OAV analysis was carried out. In general, volatile compounds exhibiting OAV ≥ 1 are deemed as important contributors to the comprehensive aroma, with higher OAV values indicating a stronger contribution [22]. In this investigation, 21 volatile constituents exhibiting OAV ≥ 1 were identified in the tea samples exposed to five distinct light intensities (Figure S3 and Table S5). The top eight volatiles with OAV > 50 in five treatments included (*E, Z*)-2,6-nonadienal (OAV: 893.58 ~ 899.69), *β*-damascenone (OAV: 467.90 ~ 840.63), *β*-ionone (OAV: 305.00 ~ 448.11), phenylethyl alcohol (OAV: 170.00 ~ 436.70), linalool (OAV: 88.02 ~ 152.93), (*E, E*)-2,4-decadienal (OAV: 92.09 ~ 99.30), (*E, E*)-2,4-nonadienal (OAV: 48.81 ~ 66.27), and phenylacetaldehyde (OAV: 50.18 ~ 75.85). Notably, *β*-ionone, (*E, E*)-2,4-nonadienal, and (*E, E*)-2,4-decadienal exhibited higher OAV values in floral aroma samples exposed to 4000 Lux and 6000 Lux compared to the other three treatments. Additionally, phenylacetaldehyde and linalool attained the highest OAV values (75.85 and 152.93, respectively) under 6000 Lux treatment. These key odorants contributed significantly towards elucidating the floral aroma of green tea, corroborating the findings of Xie et al. [23] and Tian et al. [24]. Interestingly, the OAV of nerolidol was greater than 1 only in tea samples treated with 4000 Lux, while citral exhibited an OAV > 1 exclusively in tea samples treated with 1000 Lux. Additionally, 2-methyl-propanal demonstrated the highest OAV in the tea samples treated with 1000 Lux.

#### 3.3.4. Analysis of the Correlations Between Key Volatile Components and Aroma Quality

**Figure 4:**
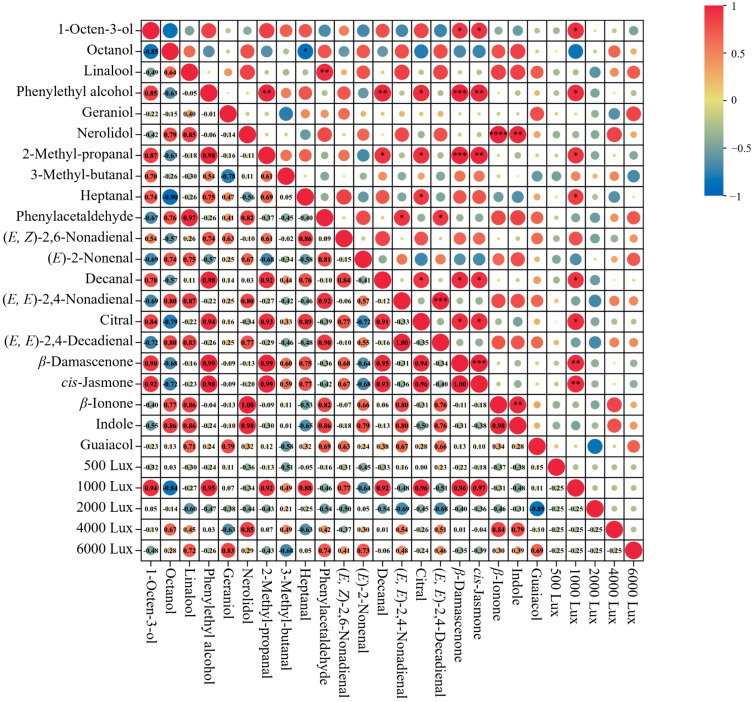
Correlation analysis between 21 key odorants with OAV > 1 and aroma quality of tea samples exposed to yellow light with different intensities. *, *p* < 0.05; **, *p* < 0.01; ***, *p* < 0.001; ****, *p* < 0.0001.

To better understand the potential intrinsic relationships between aroma quality and key volatile components, a correlation analysis was performed, with the results illustrated in Figure 4. The aroma quality of tea samples exposed to 1000 Lux showed a significant positive correlation with several volatile compounds, such as 1-octen-3-ol, *β*-damascenone, *cis*-jasmone, phenylethyl alcohol, 2-methyl-propanal, heptanal, decanal, and citral, highlighting their essential influence on the overall aroma quality.

Next, the relationships between these key odorants were further elucidated. Phenylethyl alcohol exhibited strong positive correlations with 2-methyl-propanal (r = 0.98, *p* < 0.01) and decanal (r = 0.98, *p* < 0.01). These compounds generally contribute to the honey, fruity, and floral notes [24,25]. Linalool was significantly correlated with phenylacetaldehyde (r = 0.97, *p* < 0.01), indicating their combined role in enhancing the sweet, floral, and fruity notes, which were common in high-quality tea aroma profiles [26]. 1-Octen-3-ol was positively correlated with *β*-damascenone (r = 0.90, *p* < 0.05) and cis-jasmone (r = 0.92, *p* < 0.05), which were pivotal contributors to the floral and fruity aromas [27,28]. The interactions between these ketones and alcohols likely amplify the tea’s freshness and complexity [29]. Nerolidol showed a highly significant positive correlation with *β*-ionone (r = 1.00, *p* < 0.001). Both are derived from carotenoid degradation, and this close relationship points to their synergistic role in contributing to the fruity and violet-like notes of tea [30,31,32]. Citral was significantly positively correlated with decanal (r = 0.91, *p* < 0.05), 2-methyl-propanal (r = 0.93, *p* < 0.05), and heptanal (r = 0.89, *p* < 0.05). These aldehydes are recognized for their contributions to the citrus and fruity notes, which are commonly found in green tea [24,25,31]. Additionally, a highly significant positive correlation was observed between *(E, E)*-2,4-nonadienal and *(E, E)*-2,4-decadiena (*p* < 0.001), with a correlation coefficient of *r* = 1.00. These two aldehydes, derived from fatty acids, are recognized for imparting fatty and green notes. *Cis*-Jasmone and *β*-damascenone are pivotal ketones influencing tea aroma [33]. *Cis*-Jasmone exhibited an extremely strong correlation with *β*-damascenone (r = 1.00, *p* < 0.05). Both *cis*-jasmone and *β*-damascenone were found to be significantly positively correlated with 2-methyl-propanal, decanal, and citral. It is hypothesized that these ketones and aldehydes function synergistically to enhance the complexity and richness of tea aroma.

In summary, key volatiles such as 2-methyl-propanal, phenylethyl alcohol, heptanal, decanal, citral, *cis*-jasmone, and *β*-damascenone displayed significant positive correlations with multiple pivotal odorants. These robust interactions indicate their significant contribution to the aroma development of tea samples under YLDI. Further exploration of the molecular interactions involved will enhance our understanding of the mechanisms underlying aroma formation.

#### 3.3.5. Analysis of the Metabolic Pathways of Key Volatile Compounds

By the metabolic pathways involved, the 21 key volatile components discussed above were subcategorized into four classes, as illustrated in Figure S4. According to Xie et al. [23], these comprised three carotenoid-derived volatiles (CDVs), nine fatty acid-derived volatiles (FADVs), six amino acid-derived volatiles (AADVs), and three glycoside-derived volatiles (GDVs).

**Figure 5:**
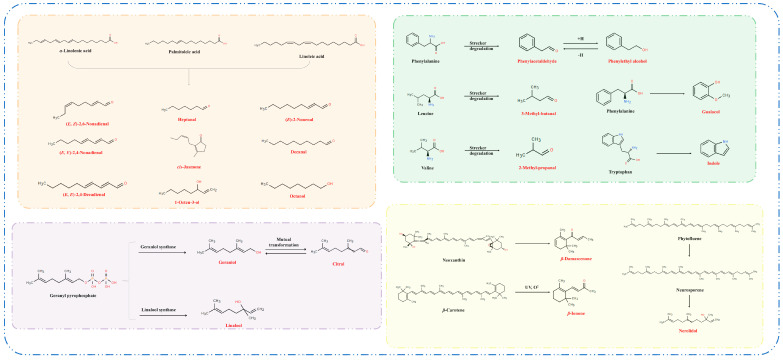
Possible formation pathways of 21 important volatile components with OAV > 1 in tea samples exposed to yellow light with different intensities.

**Figure 6:**
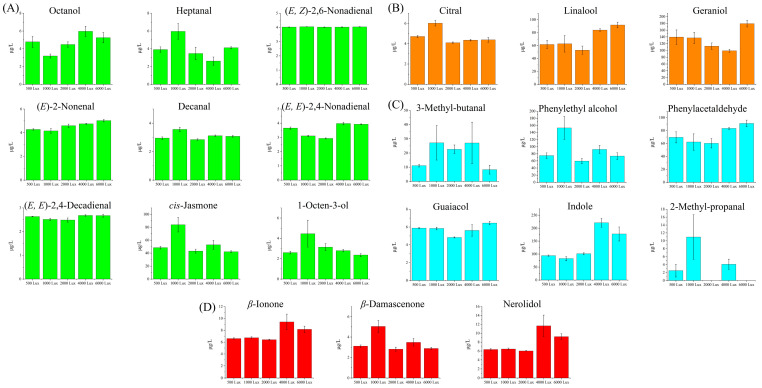
Dynamics changes of key differential volatile compounds in tea samples exposed to yellow light with different intensities. (**A**) Fatty acid-derived volatiles; (**B**) Glycoside-derived volatiles; (**C**) Amino acid-derived volatiles; (**D**) Carotenoid-derived volatiles.

FADVs are primarily derived from the oxidative degradation of unsaturated fatty acids via the lipoxygenase pathway (Figure 5). The common precursor substances in tea mainly include *α*-linolenic acid, palmitoleic acid, and linoleic acid [32]. As a crucial odorant in green tea [34], 1-octen-3-ol attained its maximum of 4.45 μg/L under 1000 Lux, then declined as the light intensity increased, mirroring the findings by He et al. [35]. As shown in Figure 6A, the trend for (*E*)-2-nonenal was opposite, with the lowest concentration (4.15 μg/L) under 1000 Lux. As previously highlighted, (*E*, *E*)-2,4-nonadienal and (*E*, *E*)-2,4-decadienal demonstrated a high correlation, both exhibiting a trend of initial decrease followed by an increase under different light intensities. *Cis*-jasmone, decanal, heptanal, and (*E*, *Z*)-2,6-nonadienal attained their highest concentrations at 1000 Lux, whereas octanol peaked at 4000 Lux.

Geraniol and linalool serve as typical GDVs, synthesized via the intermediary geranyl pyrophosphate (geranyl-PP). These enzymes, namely geraniol synthase and linalool synthase, respectively, catalyze this liberation process (Figure 5). As important aroma compounds in black tea, linalool and geraniol impart sweet, floral, fruity, rose-like, and waxy notes [23]. As shown in Figure 6B, linalool and geraniol reached the lowest concentrations under 2000 Lux (52.81 μg/L) and 4000 Lux (99.13 μg/L), respectively, and attained their highest concentrations both under 6000 Lux (91.76 μg/L and 179.25 μg/L, respectively). Citral attained its highest concentration under 1000 Lux (6.02 μg/L), noticeably higher than in other light intensities [36,37]. This divergent pattern may indicate variations in the biosynthetic regulation of these compounds. Specifically, citral is synthesized through the oxidation of geraniol and may be subject to downregulation or degradation in conditions of elevated light intensity. Conversely, linalool and geraniol appear to directly benefit from light-induced metabolic flux within the methylerythritol phosphate (MEP) pathway. Light can induce the up-regulation of the expression of MEP pathway genes, and this evidence conclusively corroborates the light-intensity-dependent metabolic channeling mechanism governing linalool and geraniol biosynthesis [35].

The AADVs, such as 3-methyl-butanal, phenylacetaldehyde, and 2-methyl-propanal, are typical Strecker aldehydes (Figure 5). They are generated via the Strecker degradation pathway, utilizing their respective amino acids of leucine, phenylalanine, and valine as precursors [32]. These AADVs exhibited distinct responses to light intensity. 2-Methyl-propanal attained its maximum (10.96 μg/L) under 1000 Lux treatment; Phenylacetaldehyde exhibited a V-shaped trend, with the highest concentration (91.03 μg/L) under 6000 Lux treatment; 3-Methyl-butanal peaked under 1000 Lux treatment, with a concentration of 27.16 μg/L (Figure 6C). Indole, another AADV, is generated from tryptophan via the action of tryptophan indole-lyase. It was reported as a pivotal floral aroma constituent in jasmine tea [32,38]. Guaiacol was reported to be derived from phenylalanine [39]. Both indole and guaiacol exhibited upward trends with the increasing of light intensity, attaining peak concentrations at 4000 Lux (220.94 μg/L) and 6000 Lux (6.44 μg/L), respectively. It is speculated that high intensity of yellow light promotes the metabolism of phenylalanine-guaiacol and tryptophan-indole [7].

CDVs such as *β*-ionone, nerolidol, and *β*-damascenone primarily originate from the oxidative degradation of carotenoids (Figure 5). Specifically, *β*-damascenone is predominantly produced through the degradation of neoxanthin, whereas *β*-ionone is generated via the oxidation of *β*-carotene. Nerolidol is derived from the photo-oxidation of phytofluene and is synthesized via the nerolidol synthase gene [32,40]. In our study, these compounds exhibited divergent responses to light intensity, with variations observed across different treatments. Nerolidol and *β*-ionone attained their peaks under 4000 Lux treatment (Figure 6D). Ni et al. [40] have found that increasing light intensity can enhance the formation of CDVs, which corroborates our findings. The interconversion of zeaxanthin, antheraxanthin, and violaxanthin, known as the xanthophyll cycle, is regulated by light. Zeaxanthin can be catalyzed into CDVs through the action of carotenoid cleavage dioxygenases. Stronger light intensity promotes the accumulation of zeaxanthin, potentially explaining the highest concentrations of nerolidol and *β*-ionone under 4000 Lux treatment. In contrast, *β*-damascenone peaked at 1000 Lux and decreased markedly at higher intensities, which may be attributed to its susceptibility to photo-oxidative degradation. These results imply that although stronger light intensity can enhance the formation of certain CDVs, it may simultaneously accelerate the loss of others, indicating that individual CDVs respond differently to light intensity.

## 4. Conclusions

This study demonstrated that the aroma quality and volatile compounds of green tea were significantly influenced by spreading under exposure to YLDI. Higher light intensities, particularly 6000 Lux, were found to enhance the overall sensory quality, with a pleasant floral aroma. Substantial variations in volatile profiles were observed in green tea exposed to YLDI via GC-E-Nose and targeted GC-MS/MS analysis. Totally, 70 volatile components were identified, with alcohols higher under 1000 Lux, aldehydes higher under 4000 Lux, and esters and alkenes higher at 6000 Lux. OAV analysis further highlighted the critical role of volatile compounds like citral, *β*-damascenone, and phenylethyl alcohol in contributing to the floral and fruity aromas, particularly in tea samples under 4000 and 6000 Lux treatments. The metabolic pathways of key aroma volatiles were also investigated, and the results demonstrated that higher light intensities facilitated the accumulation of critical aroma compounds, notably linalool and geraniol. In addition, key volatiles such as 2-methyl-propanal, phenylethyl alcohol, heptanal, decanal, citral, *cis*-jasmone, and *β*-damascenone exhibited significant positive correlations with multiple pivotal odorants, suggesting their critical role in the aroma development of tea samples under YLDI. The results provide invaluable insights for optimizing conditions to enhance the quality of green tea through light manipulation. In the future, genomics, proteomics, and other advanced techniques will be employed to elucidate the intrinsic formation mechanism of light manipulation.

## Data Availability

The original contributions presented in this study are included in the article/Supplementary Materials. Further inquiries can be directed to the corresponding authors.

## Supplementary Materials

The following supporting information can be downloaded at: https://www.mdpi.com/article/10.3390/foods14081313/s1, Figure S1: The processing processes of green tea under yellow light with different intensities; Figure S2: Volatile fingerprints on MXT-5 and MXT-1701 columns obtained from GC-E-Nose analysis; Figure S3: OAV comparison of key volatile compounds in tea samples under yellow light with different intensities; Figure S4: Classification of 21 key volatile compounds based on their pathways; Table S1: Relevant standard information of volatile compounds; Table S2: The quantification information of volatile components in this study; Table S3: Results of sensory evaluation of tea samples under yellow light with different intensities; Table S4: The contents of volatile compounds in green tea samples under yellow light with different intensities; Table S5: The OAVs of volatile compounds in green tea samples under yellow light with different intensities. References [2,23,31,41] are cited in the Supplementary Materials.
