# Supplementary material for: Decoding Light-Spreading Intensity Effects on the Sensory Quality and Volatile Compounds of Green Tea: An Integrated GC-E-Nose and Targeted Metabolomics Analysis
Source: Foods. 2025 Apr 10;14(8):1313. doi: 10.3390/foods14081313 (PMC12027006; doi:10.3390/foods14081313)
Supplement: Supplementary file 1 [file foods-14-01313-s001.zip › foods-3507786-supplementary.pdf]

Supporting Information for

Decoding Light-Spreading Intensity Effects on the Sensory Quality and Volatile Compounds of Green Tea: An Integrated GC-E-Nose and Targeted Metabolomics Analysis

Qiwei Wang [1,†], Jiajing Hu [1,†], Jiahao Tang [1,2], Xianxiu Zhou [1,3], Haibo Yuan [1], Yongwen Jiang [1], Jialing Xie [4,*] and Yanqin Yang [1,*]

[1] National Key Laboratory for Tea Plant Germplasm Innovation and Resource Utilization, Tea Research
Institute, Chinese Academy of Agricultural Sciences, Hangzhou 310008, China;
wangqiwei@tricaas.com (Q.W.); 15926789897@163.com (J.H.); tangjiahao@tricaas.com (J.T.); zhouxianxiu@tricaas.com (X.Z.); 192168092@tricaas.com (H.Y.); jiangyw@tricaas.com (Y.J.)

[2] State Key Laboratory of Tea Plant Biology and Utilization, Anhui Agricultural University,
Hefei 230036, China

[3] College of Tea Science, Yunnan Agricultural University, Kunming 650201, China

[4] Hezhou Agriculture and Rural Affairs Bureau, Hezhou 542800, China

* Correspondence: xiejialing1123@163.com (J.X.); yangyq@tricaas.com (Y.Y.)

† These authors contributed equally to this work.

Table S1. Relevant standard information of volatile compounds.

Chemicals	Purity	Brand	City	Country
1-Penten-3-ol	98%	MACKLIN	Shanghai	China
Benzyl alcohol	99.50%	MACKLIN	Shanghai	China
Benzaldehyde	98%	MACKLIN	Shanghai	China
Geraniol	85%	MACKLIN	Shanghai	China
Eugenol	99%	MACKLIN	Shanghai	China
Benzoic acid, ethyl ester	>99.5%	MACKLIN	Shanghai	China
1,2,3,5-Tetramethylbenzene	>70%	MACKLIN	Shanghai	China
(3Z)-3-Hexen-1-yl hexanoate	98%	MACKLIN	Shanghai	China
(Z)-3-Hexen-1-yl (Z)-3-hexenoate	>98%	MACKLIN	Shanghai	China
Propanoic acid, butyl ester	98%	MACKLIN	Shanghai	China
Butanoic acid, butyl ester	≥99.5%	MACKLIN	Shanghai	China
cis-Jasmone	98%	MACKLIN	Shanghai	China
(*E*)-2-Hexenol	97%	MACKLIN	Shanghai	China
D-Limonene	99%	MACKLIN	Shanghai	China
(*E*)-2-Nonenal	>95%	MACKLIN	Shanghai	China
Theaspirane	≥90%	MACKLIN	Shanghai	China
(*E, E*)-2,4-Nonadienal	>90%	MACKLIN	Shanghai	China
(*E, Z*)-2,6-Nonadienal	≥95%	MACKLIN	Shanghai	China
(*E, E*)-2,4-Decadienal	>90%	MACKLIN	Shanghai	China
β-Damascenone	98%	MACKLIN	Shanghai	China
3-Octanone	≥99.5%	MACKLIN	Shanghai	China
3-Nonen-2-one	>96%	MACKLIN	Shanghai	China
Hexanol	98%	TCI	Shanghai	China
Heptanol	98%	TCI	Shanghai	China
Heptanal	>95%	TCI	Shanghai	China
Nonanal	>95%	TCI	Shanghai	China
Hexanal	>98%	TCI	Shanghai	China
2-Methyl-propanal	>98%	TCI	Shanghai	China
β-Ionone	>95%	TCI	Shanghai	China
α-Phellandrene	>95%	TCI	Shanghai	China
(*E*)-2-hexenyl butanoate	>93%	TCI	Shanghai	China
Guaiacol	98%	ALADDIN	Shanghai	China
Hexanoic acid, ethyl ester	>99%	ALADDIN	Shanghai	China
Phenylacetaldehyde	>99%	ALADDIN	Shanghai	China
1-Octen-3-ol	98%	ALADDIN	Shanghai	China

3-Methyl-butanal	>99%	ALADDIN	Shanghai	China
2-Methyl-naphthalene	>97%	ALADDIN	Shanghai	China
Carene	>90%	ALADDIN	Shanghai	China
cis-3-Hexenyl acetate	98%	ALADDIN	Shanghai	China
Acetic acid, butyl ester	>98%	ALADDIN	Shanghai	China
Nerol	≥98%	ALADDIN	Shanghai	China
Nerolidol	97%	ALADDIN	Shanghai	China
2,4-Dimethyl-1-heptene	≥98%	MERYER	Shanghai	China
Geranyl acetone	97%	MERYER	Shanghai	China
Butanoic acid, 2-phenylethyl ester	98%	MERYER	Shanghai	China
(*E*)-2-Hexene-1-ol hexanoate	97%	MERYER	Shanghai	China
Octanol	>99.5%	ACMEC	Shanghai	China
(*3Z*)-3-Hexen-1-yl benzoate	97%	ACMEC	Shanghai	China
(*E*)-2-Decenal	95%	ACMEC	Shanghai	China
Hexyl isovalerate	98%	ACMEC	Shanghai	China
Phenylethyl alcohol	>99%	J&K Scientific	Beijing	China
Linalool	98%	J&K Scientific	Beijing	China
Decanal	97%	J&K Scientific	Beijing	China
Butanoic acid, hexyl ester	>98%	J&K Scientific	Beijing	China
Hexanoic acid, hexyl ester	99%	J&K Scientific	Beijing	China
Geraniol	≥98%	Yuanye	Shanghai	China
Butanoic acid, phenylmethyl ester	>97.5%	Yuanye	Shanghai	China
(*E*)-2-Hexenal	≥99%	Yuanye	Shanghai	China
Nonanol	99.50%	Yingxin	Shanghai	China
Citral	≥98%	Yingxin	Shanghai	China
Ethyl salicylate	≥98%	Yingxin	Shanghai	China
Safranal	>90%	Psaitong	Beijing	China
Coumarin	≥99.5%	APExBIO	Houston	US
δ-Cadinene	>95%	D&B	Shanghai	China
cis-3-Hexenyl salicylate	97%	RHAWN	Shanghai	China
cis-3-Hexenyl-α-methylbutyrate	96%	HEOWNS	Tianjin	China
β-Myrcene	98%	NATUREWILL	chengdu	China
6-Methyl-5-hepten-2-one	98%	ThermoFisher	Waltham	US
Indole	>97%	SOLARBIO	Beijing	China
(*E, E*)-2,4-Heptadienal	≥90%	TRC	Toronto	Canada

Table S2 The quantification information of volatile components in this study.

RT (min)	Compounds	Qualitative ion pairs	Quantitative ion pairs	Calibration curves	R^2
1.92	2-Methyl-propanal	72→43	72→57	y=4304.98x+21697.81	0.993
2.55	3-Methyl-butanal	71→41	71→43	y=2449.46x+3364.93	0.995
2.98	1-Penten-3-ol	72→42	72→57	y=4167.53x+6616.27	0.994
6.02	Hexanal	72→44	72→43	y=43567.19x-52483.44	0.998
6.81	Acetic acid, butyl ester	56→54.2	56→41.2	y=225117.751x+48965.850	0.994
7.66	2,4-Dimethyl-1-heptene	126→70	126→83	y=22827.55x-11976.62	0.999
8.27	(*E*)-2-Hexenal	83→81.5	83→55	y=15836.338x+37526.248	0.997
8.79	(*E*)-2-Hexenol	91→61	91→65	y=3436.91x+12977.33	0.999
8.95	Hexanol	84→56	84→69	y=2141.12x-1072.38	0.999
10.36	Heptanal	70→41	70→55	y=98737.53x+297533.43	0.997
10.80	Propanoic acid, butyl ester	57.1→42	57.1→41	y=86096.769x+1442.054	0.999
12.88	Benzaldehyde	77→74	77→50	y=2591.32x+1734.97	0.998
13.37	Heptanol	70→42	70→55	y=184429.37x+69698.65	0.997
14.05	1-Octen-3-ol	99→71	99→43	y=10159.90x-4409.53	0.999
14.13	6-Methyl-5-hepten-2-one	108.1→93	108.1→79	y=1401.98x-84.95	0.998
14.15	3-Octanone	108.1→90.8	108.1→92.9	y=15763.45x+6793.69	0.993
14.31	*β*-Myrcene	93→77	93→91.2	y=90325.128x+5463.318	0.998
14.67	Butanoic acid, butyl ester	89→59	89→61.8	y=6741.602x-549.925	0.999
14.83	Hexanoic acid, ethyl ester	88→70.2	88→61	y=173031.921x-9315.301	0.999
14.94	*α*-Phellandrene	93.1→77	93.1→91	y=81896.337x+4966.399	0.998

15.10	Carene	93.1→77	93.1→91	y=9897.855x+10612.957	0.993
15.27	(*E, E*)-2,4-Heptadienal	110→95	110→81	y=47015.80x-145174.18	0.991
15.92	*D*-Limonene	93→77	93→91.2	y=124798.82x-92419.93	0.999
16.01	Benzyl alcohol	108→80	108→93	y=18161.41x-13410.60	0.999
16.40	Phenylacetaldehyde	120→117	120→105	y=3445.61x+14638.88	0.999
17.80	Octanol	84.1→69	84.1→56	y=40372.056x-2102.621	0.998
18.30	Guaiacol	109→78	109→80	y=736.65x-3150.02	0.999
19.09	Linalool	93→77	93→91.2	y=27284.49x+123509.14	0.999
19.16	Nonanal	98→56	98→69	y=7247.74x-3304.40	0.999
19.53	Phenylethyl alcohol	122→97	122→92	y=3924.47x+2096.28	0.999
20.36	3-Nonen-2-one	125→92	125→94	y=22.91x+10.72	0.996
20.72	1,2,3,5-Tetramethylbenzene	134→108	134→89.8	y=47.878x-22.985	0.995
21.06	(*E, Z*)-2,6-Nonadienal	70→55	70→42	y=71904.86x-283393.69	0.998
21.43	(*E*)-2-Nonenal	83→41	83→55	y=94344.73x-335643.29	0.997
21.62	Benzoic acid, ethyl ester	122→109	122→120	y=1664.087x+3572.989	0.996
22.18	Nonanol	97→69	97→55	y=93780.72x-293297.69	0.995
22.54	Butanoic acid, hexyl ester	89.1→58.5	89.1→43	y=33852.109x-20168.163	0.999
22.77	(*E*)-2-Hexenyl butanoate	71.1→41	71.1→43	y=217866.566x+335708.510	0.990
22.80	Safranal	107.1→79	107.1→90.8	y=7029.397x+361.719	0.997
23.15	Decanal	112→69	112→70	y=38075.74x-89821.97	0.994
23.20	*cis*-3-Hexenyl acetate	89→84.3	89→63.3	y=336.825x-25.243	0.999
23.52	(*E, E*)-2,4-Nonadienal	81→79	81→53	y=6069.34x-17269.68	0.996

23.89	*cis*-3-Hexenyl-α-methylbutyrate	82.1→54	82.1→66.8	y=333191.283x-5847.491	0.999
24.05	Nerol	93→77	93→91	y=2660.503x+420.402	0.991
24.11	Hexyl isovalerate	85→43.2	85→57	y=280218.753x+6945.230	0.999
24.78	Geraniol	93→77	93→91	y=11028.11x-41301.85	0.998
24.80	(*E*)-2-Decenal	70→42.2	70→55	y=112723.849x-62877.371	0.999
25.30	Citral	84→41	84→56	y=11094.96x-30499.48	0.995
25.34	Ethyl salicylate	120.1→88	120.1→92	y=114663.174x-174621.204	0.998
25.70	Indole	117→115	117→90	y=3299.50x-15025.98	0.999
26.01	Theaspirane	138→109	138→96	y=201627.62x-143935.19	0.998
26.43	2-Methyl-naphthalene	115→96.6	115→73	y=115.964x-263.021	0.998
27.31	(*E, E*)-2,4-decadienal	81→79	81→53	y=13670.45x-32470.06	0.994
28.05	Butanoic acid, phenylmethyl ester	108.1→66	108.1→78.8	y=121.554x-233.346	0.998
28.64	Eugenol	149→125	149→119	y=441.331x+362.413	0.999
28.75	Geranic acid	100→79.8	100→81.7	y=442.639x-1075.339	0.993
28.94	(*Z*)-3-Hexen-1-yl (*Z*)-3-hexenoate	82.1→73	82.1→55.2	y=19337.991x-14757.775	0.999
29.20	(*3Z*)-3-Hexen-1-yl hexanoate	99→57	99→71	y=87922.59x-8886.50	0.996
29.41	Hexanoic acid, hexyl ester	117.1→90.8	117.1→86	y=904.385x-291.118	0.997
29.57	*β*-Damascenone	123→89	123→95	y=10991.77x-5539.93	0.999
29.70	*cis*-Jasmone	164→150	164→135	y=3667.95x-8130.43	0.999
29.76	(*E*)-2-Hexene-1-ol hexanoate	99.1→62	99.1→82.3	y=29.849x-16.387	0.994
30.52	Coumarin	146→135.4	146→140.7	y=20.2429x-47.950	0.994
31.14	Butanoic acid, 2-phenylethyl ester	104.1→84.3	104.1→80.4	y=20.182x-6.857	0.999

31.40	Geranyl acetone	136→108	136→121	y=34497.13x-58944.40	0.995
32.30	*β*-Ionone	177→107	177→121	y=976.25x-5819.20	0.994
33.48	*δ*-Cadinene	161.2→131	161.2→105.1	y=36443.296x-59.783	0.999
33.85	Nerolidol	93→77	93→91	y=27713.67x-146729.74	0.997
34.89	(*3Z*)-3-Hexen-1-yl benzoate	105→78.7	105→74.8	y=430.395x+275.845	0.998
38.03	*cis*-3-Hexenyl salicylate	120→96.7	120→92	y=20198.400x+6182.540	0.999

Table S3 Results of sensory evaluation of tea samples under yellow light with different intensities.

	500 Lux		1000 Lux		2000 Lux		4000 Lux		6000 Lux	
	Comments	Scores	Comments	Scores	Comments	Scores	Comments	Scores	Comments	Scores
Appearance	Wiry, deep green, light evenly	88.17±0.29b	Wiry, deep green, evenly	89.17±0.76b	Light wiry, deep green, evenly	88.17±0.76b	Light wiry, black green, light evenly	88.33±1.53b	Wiry, deep green, evenly	91.17±1.04a
Liquor color	Tender yellow, light bright	87.33±1.15d	Tender green, bright	91.67±1.53ab	Tender yellowish green, bright	88.33±0.58cd	Tender yellowish green, bright	89.83±1.04bc	Yellowish green, bright	92.33±0.58a
Aroma	Tender chestnut-like aroma	90.00±1.00c	Chestnut-like aroma with floral aroma	92.17±0.29b	Tender chestnut-like aroma	90.33±0.76c	Floral aroma	91.67±0.58b	Floral aroma, strong and lasting	93.67±0.58a
Taste	Light mellow and brisk	88.33±1.53d	Fresh and floral taste	91.33±0.58ab	Mellow	89.00±1.00cd	Mellow and normal	90.00±1.00bc	Floral taste, heavy and mellow	93.00±1.00a
Infused leaf	Yellowish green, light evenly, bright	88.33±0.58b	Tender yellowish green, evenly, bright	90.00±0.87ab	Tender green, light evenly, green and bright	88.33±0.58b	Yellowish green, evenly	89.67±1.53ab	Tender green, evenly, bright	91.50±1.32a
Total Quality score		88.61±0.25d		90.90±0.59b		88.99±0.31d		89.95±0.73c		92.49±0.30a

Note: Different letters (a-d) in the same row indicated the significance of tea samples under yellow light with different intensities ($p < 0.05$).

Table S4 The contents of volatile compounds in green tea samples under yellow light with different intensities.

Compounds	Contents (μg/L)					*p*-value	VIP
	500 Lux	1000 Lux	2000 Lux	4000 Lux	6000 Lux		
2-Methyl-propanal	2.49±1.60b	10.96±5.62a	0.00±0.00b	4.09±1.27b	0.00±0.00b	0.003	0.932
3-Methyl-butanal	11.09±0.94ab	27.16±12.12a	22.60±3.04ab	27.06±14.65a	8.263.08b	0.063	1.014
1-Penten-3-ol	11.06±2.35bc	28.66±13.53a	22.96±3.17ab	17.60±3.02ab	1.36±0.44c	0.004	0.989
Hexanal	1.25±0.02ab	1.22±0.01b	1.26±0.02a	1.26±0.01a	1.26±0.02a	0.022	0.866
Acetic acid, butyl ester	1.46±0.08ab	1.08±0.03c	1.39±0.14b	1.48±0.06ab	1.59±0.10a	0.000	0.784
2,4-Dimethyl-1-heptene	0.53±0.00a	0.53±0.00a	0.53±0.00a	0.53±0.00a	0.53±0.00a	0.041	0.732
(*E*)-2-Hexenal	1.00±0.89a	0.14±0.23b	0.00±0.00b	0.00±0.00b	0.00±0.00b	0.082	1.401
(*E*)-2-Hexenol	0.59±0.57b	0.00±0.00b	0.29±0.28b	1.68±0.51a	0.25±0.20b	0.002	1.161
Hexanol	9.33±0.95b	13.29±1.56a	10.54±0.78b	8.87±0.11b	12.28±0.25a	0.001	1.206
Heptanal	3.91±0.30b	5.96±0.88a	3.46±0.70bc	2.62±0.46c	4.11±0.12b	0.000	0.964
Propanoic acid, butyl ester	0.52±0.02a	0.38±0.01b	0.45±0.09ab	0.44±0.01ab	0.47±0.02a	0.028	1.063
Benzaldehyde	2.92±0.47bc	5.89±0.45a	2.68±0.67c	3.81±0.65b	3.26±0.39bc	0.000	0.940
Heptanol	0.00±0.00a	0.01±0.02a	0.00±0.00a	0.07±0.07a	0.06±0.10a	0.407	0.600
1-Octen-3-ol	2.59±0.14b	4.45±1.32a	3.13±0.35b	2.78±0.12b	2.35±0.15b	0.014	0.759
6-Methyl-5-hepten-2-one	2.38±0.24b	0.37±0.19c	3.33±0.98a	2.04±0.38b	3.53±0.34a	0.000	1.082
3-Octanone	0.00±0.00b	0.00±0.00b	0.00±0.00b	0.29±0.07a	0.01±0.02b	0.000	1.224
β-Myrcene	2.49±0.09a	2.37±0.03a	2.49±0.20a	2.59±0.23a	2.50±0.05a	0.470	0.541
Butanoic acid, butyl ester	0.10±0.00c	0.10±0.00c	0.11±0.00b	0.13±0.00a	0.13±0.01a	0.000	1.026
Hexanoic acid, ethyl ester	0.08±0.00b	0.08±0.00b	0.08±0.00b	0.10±0.00a	0.10±0.00a	0.000	0.889
α-Phellandrene	2.46±0.07a	2.37±0.11a	2.47±0.22a	2.37±0.14a	2.47±0.04a	0.715	0.565
Carene	24.47±3.86a	22.97±2.66a	21.07±1.78a	24.28±1.62a	26.32±4.10a	0.343	0.842
(*E, E*)-2,4-Heptadienal	3.54±0.05c	4.35±0.24a	3.82±0.05b	3.65±0.12bc	3.50±0.02c	0.000	0.913
D-Limonene	1.27±0.04b	1.31±0.04b	1.28±0.01b	1.66±0.08a	1.25±0.03b	0.000	1.275
Benzyl alcohol	1.05±0.03b	1.06±0.04b	1.06±0.01b	1.33±0.06a	1.06±0.01b	0.000	1.216
Phenylacetaldehyde	69.42±8.5bc	62.20±12.64c	60.21±7.18c	83.32±1.54ab	91.03±5.15a	0.003	0.841
Octanol	4.78±0.61b	3.18±0.23c	4.48±0.30b	5.96±0.56a	5.25±0.58ab	0.000	0.825
Guaiacol	5.87±0.08b	5.84±0.15b	4.83±0.05c	5.62±0.64b	6.44±0.20a	0.001	1.321

Linalool	61.56±6.45b	62.71±12.78b	52.81±6.41b	83.90±2.04a	91.76±4.45a	0.000	0.911
Nonanal	0.62±0.01b	0.70±0.04a	0.62±0.02b	0.68±0.01a	0.60±0.01b	0.001	1.046
Phenylethyl alcohol	74.92±7.65bc	152.85±32.10a	59.50±7.42c	92.29±10.72b	73.65±9.07bc	0.000	0.942
3-Nonen-2-one	0.86±0.99b	0.00±0.00b	0.03±0.04b	0.10±0.08b	2.10±1.00a	0.009	1.055
1,2,3,5-Tetramethylbenzene	0.67±0.05ab	0.54±0.02b	0.93±0.26a	0.76±0.20ab	0.88±0.12a	0.066	1.150
(*E, Z*)-2,6-Nonadienal	4.03±0.01a	4.05±0.01a	4.02±0.01a	4.02±0.02a	4.04±0.01a	0.119	0.842
(*E*)-2-Nonenal	4.27±0.06c	4.15±0.19c	4.59±0.14b	4.75±0.04b	5.02±0.10a	0.000	1.110
Benzoic acid, ethyl ester	9.74±0.58c	8.90±0.89c	8.74±0.01c	11.92±0.38b	15.45±1.30a	0.000	0.973
Nonanol	3.57±0.02b	3.78±0.05a	3.67±0.14ab	3.70±0.07ab	3.73±0.06ab	0.078	1.036
Butanoic acid, hexyl ester	0.66±0.01c	0.75±0.02b	0.66±0.01c	0.76±0.02b	0.79±0.02a	0.000	1.083
(*E*)-2-hexenyl butanoate	0.77±0.56b	0.00±0.00b	0.37±0.32b	1.65±0.45a	0.48±0.26b	0.008	1.112
Safranal	2.32±0.08a	1.30±0.16d	1.73±0.10c	2.20±0.15ab	2.02±0.12b	0.000	1.233
Decanal	2.95±0.09bc	3.56±0.15a	2.85±0.05c	3.12±0.06b	3.08±0.06b	0.000	0.993
cis-3-Hexenyl acetate	0.94±0.14a	0.62±0.13a	0.65±0.44a	0.75±0.11a	0.76±0.18a	0.516	0.919
(*E, E*)-2,4-Nonadienal	3.65±0.08b	3.11±0.05c	2.93±0.03d	3.98±0.09a	3.93±0.03a	0.000	1.225
cis-3-Hexenyl-α-methylbutyrate	1.71±0.15c	2.39±0.57c	1.98±0.21c	3.38±0.39b	4.61±0.47a	0.000	1.054
Nerol	11.73±2.46bc	9.69±2.22c	16.98±2.33a	14.10±1.03ab	8.04±2.63c	0.004	1.231
hexyl isovalerate	0.57±0.08c	0.79±0.13b	0.57±0.06c	1.16±0.09a	1.19±0.05a	0.000	1.001
Geraniol	139.40±21.26b	137.20±16.64bc	112.72±9.60cd	99.13±4.41d	179.25±9.94a	0.000	1.298
(*E*)-2-Decenal	0.72±0.02a	0.76±0.02a	0.74±0.05a	0.74±0.02a	0.73±0.01a	0.539	0.627
Citral	4.71±0.10b	6.02±0.27a	4.10±0.07c	4.35±0.08c	4.38±0.24c	0.000	1.031
Ethyl salicylate	1.58±0.00b	1.73±0.02a	1.57±0.00bc	1.57±0.00bc	1.56±0.00c	0.000	0.885
Indole	93.80±2.61c	82.51±7.90c	101.17±3.61c	220.94±16.93a	177.57±27.17b	0.000	0.945
Theaspirane	0.78±0.01bc	0.76±0.01c	0.79±0.01b	0.85±0.02a	0.79±0.01b	0.000	1.063
2-Methyl-naphthalene	4.53±0.43b	2.86±0.33c	3.86±0.13bc	5.67±0.44a	6.57±1.14a	0.000	0.802
(*E, E*)-2,4-decadienal	2.63±0.01a	2.51±0.05b	2.49±0.09b	2.68±0.03a	2.67±0.06a	0.003	1.123
Butanoic acid, phenylmethyl ester	22.34±0.83ab	10.63±2.19d	16.85±1.50c	19.99±0.81b	23.13±2.14a	0.000	1.004

Eugenol	1.38±0.04b	1.49±0.29b	1.40±0.07b	2.08±0.16a	1.75±0.28ab	0.007	0.921
Geranic acid	3.10±0.08c	3.69±0.36c	3.30±0.25c	4.94±0.69b	6.34±0.73a	0.000	1.020
(*Z*)-3-Hexen-1-yl (*Z*)-3-hexenoate	1.63±0.10c	3.09±0.86b	1.69±0.24c	3.20±0.56b	5.98±1.02a	0.000	1.124
(*3Z*)-3-Hexen-1-yl hexanoate	16.95±2.05d	30.21±2.68c	12.63±2.72d	46.94±4.42b	53.26±3.51a	0.000	1.020
Hexanoic acid, hexyl ester	0.60±0.02b	0.76±0.10b	0.60±0.07b	1.14±0.13b	1.78±0.60a	0.002	0.960
β-Damascenone	3.10±0.12bc	5.04±0.59a	2.81±0.20c	3.48±0.37b	2.88±0.10bc	0.000	0.938
cis-Jasmone	48.38±2.10b	83.89±11.22a	43.04±3.12b	52.73±6.68b	42.09±1.37b	0.000	0.919
(*E*)-2-Hexene-1-ol hexanoate	1.01±0.10bc	2.04±0.52a	0.75±0.10c	1.41±0.32b	1.24±0.01bc	0.002	0.964
Coumarin	2.57±0.05a	2.45±0.03a	2.54±0.09a	2.73±0.34a	3.20±0.62a	0.102	0.750
Butanoic acid, 2-phenylethyl ester	0.87±0.58a	0.57±0.19a	0.28±0.24a	0.61±0.22a	0.50±0.06a	0.315	1.217
Geranyl acetone	2.46±0.06c	3.28±0.09a	2.57±0.08bc	2.69±0.09b	2.59±0.02bc	0.000	0.953
β-Ionone	6.60±0.13c	6.74±0.17c	6.40±0.10c	9.41±1.32a	8.16±0.54b	0.001	0.937
δ-Cadinene	0.11±0.01ab	0.05±0.01b	0.17±0.07a	0.15±0.06ab	0.22±0.11a	0.054	0.900
Nerolidol	6.34±0.21c	6.45±0.23c	6.01±0.13c	11.66±2.43a	9.23±0.64b	0.000	0.949
(*3Z*)-3-Hexen-1-yl benzoate	0.00±0.00b	0.55±0.19a	0.00±0.00b	0.00±0.00b	0.00±0.00b	0.000	0.860
cis-3-Hexenyl salicylate	0.00±0.00b	0.10±0.05a	0.00±0.00b	0.00±0.00b	0.00±0.00b	0.001	0.823

Note: Values with different letters (a–d) in the same row indicated significant differences ($p < 0.05$); VIP represented variable importance in the projection.

Table S5 The OAVs of volatile compounds in green tea samples under yellow light with different intensities.

Compounds	OTs (μg/L)	OAVs				
		500 Lux	1000 Lux	2000 Lux	4000 Lux	6000 Lux
2-Methyl-propanal	0.49 [A]	5.07	22.37	0.00	8.36	0.00
3-Methyl-butanal	1.1 [B]	10.08	24.69	20.54	24.60	7.50
1-Penten-3-ol	400 [C]	0.03	0.07	0.06	0.04	0.00
Hexanal	4.5 [C]	0.28	0.27	0.28	0.28	0.28
Acetic acid, butyl ester	100 [B]	0.01	0.01	0.01	0.01	0.02
2,4-Dimethyl-1-heptene	n.f.	/	/	/	/	/
(*E*)-2-Hexenal	17 [C]	0.06	0.01	0.00	0.00	0.00
(*E*)-2-Hexenol	232 [C]	0.00	0.00	0.00	0.01	0.00
Hexanol	500 [C]	0.02	0.03	0.02	0.02	0.02
Heptanal	0.9 [C]	4.35	6.62	3.85	2.91	4.57
Propanoic acid, butyl ester	200 [C]	0.00	0.00	0.00	0.00	0.00
Benzaldehyde	350 [C]	0.01	0.02	0.01	0.01	0.01
Heptanol	400 [C]	0.00	0.00	0.00	0.00	0.00
1-Octen-3-ol	1 [C]	2.59	4.45	3.13	2.78	2.35
6-Methyl-5-hepten-2-one	50 [C]	0.05	0.01	0.07	0.04	0.07
3-Octanone	21.4 [B]	0.00	0.00	0.00	0.01	0.00
β-Myrcene	15 [C]	0.17	0.16	0.17	0.17	0.17
Butanoic acid, butyl ester	100 [B]	0.00	0.00	0.00	0.00	0.00
Hexanoic acid, ethyl ester	5 [C]	0.02	0.02	0.02	0.02	0.02
α-Phellandrene	160 [C]	0.02	0.01	0.02	0.01	0.02
Carene	4000 [C]	0.01	0.01	0.01	0.01	0.01
(*E, E*)-2,4-Heptadienal	10000 [C]	0.00	0.00	0.00	0.00	0.00
D-Limonene	34 [C]	0.04	0.04	0.04	0.05	0.04
Benzyl alcohol	100 [C]	0.01	0.01	0.01	0.01	0.01
Phenylacetaldehyde	1.2 [C]	57.85	51.83	50.18	69.43	75.85
Octanol	3 [B]	1.59	1.06	1.49	1.99	1.75
Guaiacol	0.84 [A]	6.99	6.95	5.75	6.69	7.67
Linalool	0.6 [C]	102.6	104.5	88.02	139.83	152.93
Nonanal	2.8 [A]	0.22	0.25	0.22	0.24	0.21
Phenylethyl alcohol	0.35 [C]	214.05	436.70	170.00	263.69	210.43
3-Nonen-2-one	800 [B]	0.00	0.00	0.00	0.00	0.00
1,2,3,5-Tetramethylbenzene	n.f.	/	/	/	/	/
(*E, Z*)-2,6-Nonadienal	0.0045 [A]	894.49	899.69	893.58	894.20	897.98
(*E*)-2-Nonenal	0.4 [A]	10.67	10.37	11.47	11.87	12.55

Benzoic acid, ethyl ester	56 [C]	0.17	0.16	0.16	0.21	0.28
Nonanol	45.5 [C]	0.08	0.08	0.08	0.08	0.08
Butanoic acid, hexyl ester	250 [C]	0.00	0.00	0.00	0.00	0.00
(*E*)-2-Hexenyl butanoate	3130 [D]	0.00	0.00	0.00	0.00	0.00
Safranal	3 [D]	0.77	0.43	0.58	0.73	0.67
Decanal	2.6 [C]	1.14	1.37	1.09	1.20	1.18
cis-3-Hexenyl acetate	n.f.	/	/	/	/	/
(*E, E*)-2,4-Nonadienal	0.06 [A]	60.90	51.80	48.81	66.27	65.42
cis-3-Hexenyl-α-methylbutyrate	n.f.	/	/	/	/	/
Nerol	290 [C]	0.04	0.03	0.06	0.05	0.03
hexyl isovalerate	22 [C]	0.03	0.04	0.03	0.05	0.05
Geraniol	7.5 [C]	18.59	18.29	15.03	13.22	23.90
(*E*)-2-Decenal	2.7 [C]	0.27	0.28	0.27	0.27	0.27
Citral	5 [A]	0.94	1.20	0.82	0.87	0.88
Ethyl salicylate	84 [C]	0.02	0.02	0.02	0.02	0.02
Indole	11 [A]	8.53	7.50	9.20	20.09	16.14
Theaspirane	n.f.	/	/	/	/	/
2-Methyl-naphthalene	10 [C]	0.45	0.29	0.39	0.57	0.66
(*E, E*)-2,4-decadienal	0.027 [A]	97.59	93.12	92.09	99.30	99.01
Butanoic acid, phenylmethyl ester	376 [C]	0.06	0.03	0.04	0.05	0.06
Eugenol	150 [C]	0.01	0.01	0.01	0.01	0.01
Geranic acid	n.f.	/	/	/	/	/
(*Z*)-3-Hexen-1-yl (*Z*)-3-hexenoate	n.f.	/	/	/	/	/
(*3Z*)-3-Hexen-1-yl hexanoate	781 [D]	0.02	0.04	0.02	0.06	0.07
Hexanoic acid, hexyl ester	6400 [C]	0.00	0.00	0.00	0.00	0.00
β-Damascenone	0.006 [A]	516.85	840.63	467.90	579.79	479.47
cis-Jasmone	7 [C]	6.91	11.98	6.15	7.53	6.01
(*E*)-2-Hexene-1-ol hexanoate	195 [C]	0.01	0.01	0.00	0.01	0.01
Coumarin	11 [C]	0.23	0.22	0.23	0.25	0.29
Butanoic acid, 2-phenylethyl ester	376 [C]	0.00	0.00	0.00	0.00	0.00
Geranyl acetone	60 [C]	0.04	0.05	0.04	0.04	0.04
β-Ionone	0.021 [D]	314.18	320.76	305.0	448.11	388.57
δ-Cadinene	1.5 [C]	0.07	0.03	0.11	0.10	0.15
Nerolidol	10 [C]	0.63	0.65	0.60	1.17	0.92
(*3Z*)-3-Hexen-1-yl benzoate	500 [C]	0.00	0.00	0.00	0.00	0.00

cis-3-Hexenyl salicylate	13 [C]	0.00	0.01	0.00	0.00	0.00

Note: OT represented the odor threshold value of the compound in water. All odor thresholds were obtained from: A, [2]; B, [41]; C, [23]; D, [31]. “n.f.”, data was not found in the literatures.

The odor thresholds of volatile components found in the following literatures:

2. Zhai, X.; Zhang, L.; Granvogl, M.; Ho, C.; Wan, X. Flavor of Tea (Camellia sinensis): A Review on Odorants and Analytical Techniques. *Compr. Rev. Food Sci. Food Saf.* **2022**, *21*, 3867–3909. https://doi.org/10.1111/1541-4337.12999.

23. Xie, J.; Wang, L.; Deng, Y.; Yuan, H.; Zhu, J.; Jiang, Y.; Yang, Y. Characterization of the Key Odorants in Floral Aroma Green Tea Based on GC-E-Nose, GC-IMS, GC-MS and Aroma Recombination and Investigation of the Dynamic Changes and Aroma Formation during Processing. *Food Chem.* **2023**, *427*, 136641. https://doi.org/10.1016/j.foodchem.2023.136641.

31. Guo, X.; Ho, C.-T.; Wan, X.; Zhu, H.; Liu, Q.; Wen, Z. Changes of Volatile Compounds and Odor Profiles in Wuyi Rock Tea during Processing. *Food Chem.* **2021**, *341*, 128230. https://doi.org/10.1016/j.foodchem.2020.128230.

41. Van Gemert, L.J. *ODOUR THRESHOLDS—Compilations of Odour Threshold Values in Air, Water and Other Media*; Oliemans Punter& Partners BV: Zeist, The Netherlands, 2011.

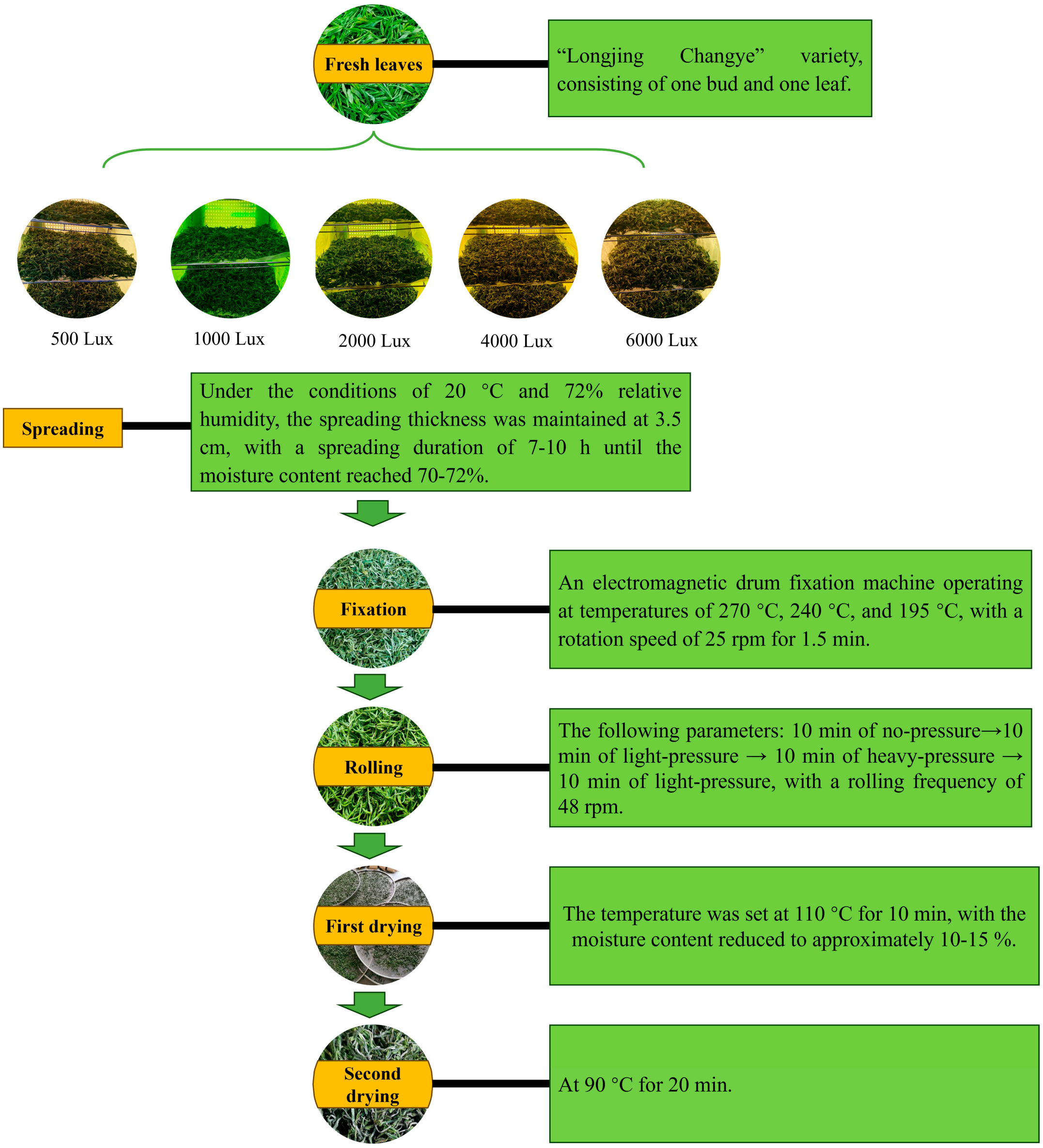

Fig. S1 The processing processes of green tea under yellow light with different intensities.

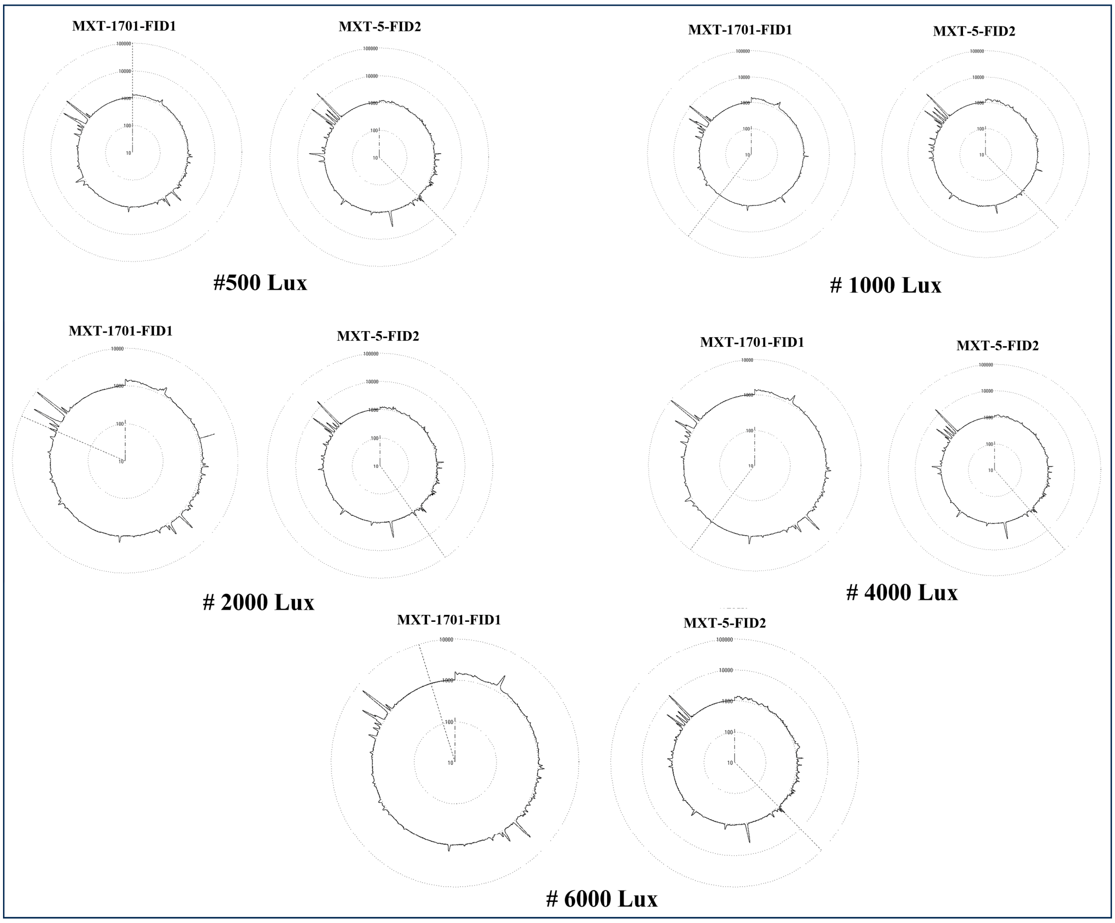

Fig. S2 Volatile fingerprints on MXT-5 and MXT-1701 columns obtained from GC-E-Nose analysis.

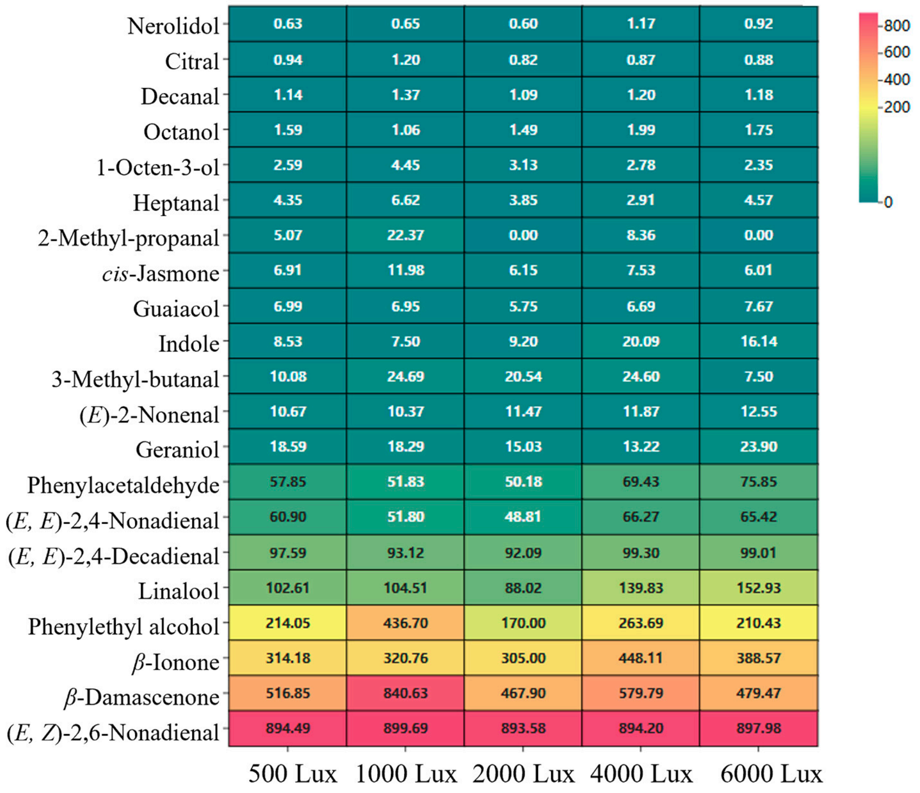

Fig. S3 OAV comparison of key volatile compounds in tea samples under yellow light with different intensities.

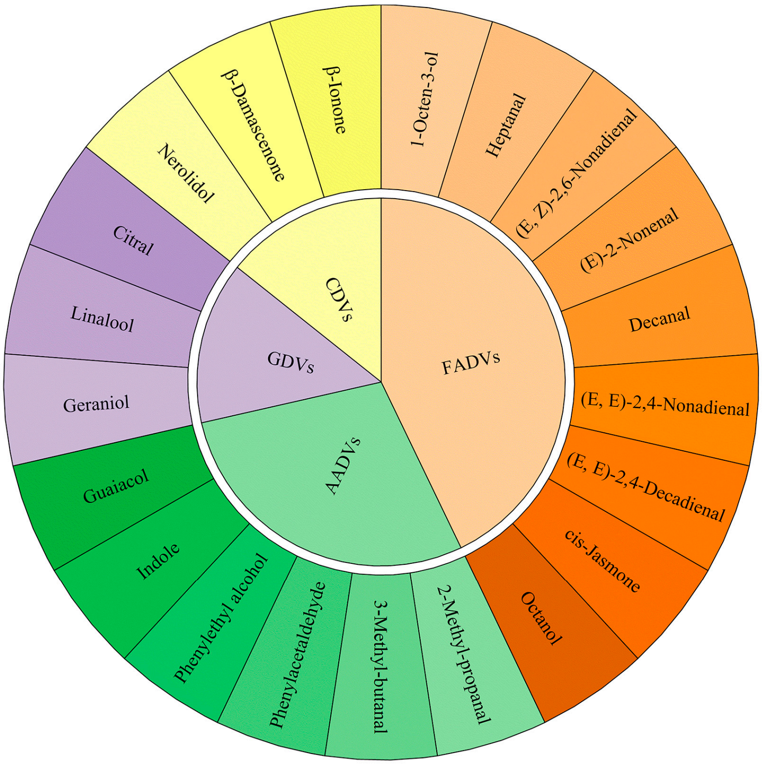

Fig. S4 Classification of 21 key volatile compounds based on their pathways.
